# Thermostabilisation of an Agonist-Bound Conformation of the Human Adenosine A_2A_ Receptor

**DOI:** 10.1016/j.jmb.2011.03.075

**Published:** 2011-06-10

**Authors:** Guillaume Lebon, Kirstie Bennett, Ali Jazayeri, Christopher G. Tate

**Affiliations:** 1 MRC Laboratory of Molecular Biology, Hills Road, CB2 0QH Cambridge, UK; 2 Heptares Therapeutics, BioPark, Broadwater Road, Welwyn Garden City AL7 3AX, UK

**Keywords:** GPCR, G-protein-coupled receptor, A_2A_R, A_2A_ receptor, T4L, T4 lysozyme, β_1_AR, β_1_ adrenoceptor, TM, transmembrane region, DM, *n*-decyl-β-d-maltopyranoside, CHO, Chinese hamster ovary, NG, nonylglucoside, EDTA, ethylenediaminetetraacetic acid, FBS, fetal bovine serum, WT, wild type, DDM, *n*-dodecyl-β-d-maltopyranoside, NECA, 5'-N-ethylcarboxamidoadenosine, conformational thermostabilisation, G-protein-coupled receptors, membrane protein, structure

## Abstract

The adenosine A_2A_ receptor (A_2A_R) is a G-protein-coupled receptor that plays a key role in transmembrane signalling mediated by the agonist adenosine. The structure of A_2A_R was determined recently in an antagonist-bound conformation, which was facilitated by the T4 lysozyme fusion in cytoplasmic loop 3 and the considerable stabilisation conferred on the receptor by the bound inverse agonist ZM241385. Unfortunately, the natural agonist adenosine does not sufficiently stabilise the receptor for the formation of diffraction-quality crystals. As a first step towards determining the structure of A_2A_R bound to an agonist, the receptor was thermostabilised by systematic mutagenesis in the presence of the bound agonist [^3^H]5'-N-ethylcarboxamidoadenosine (NECA). Four thermostabilising mutations were identified that when combined to give mutant A_2A_R-GL26, conferred a greater than 200-fold decrease in its rate of unfolding compared to the wild-type receptor. Pharmacological analysis suggested that A_2A_R-GL26 is stabilised in an agonist-bound conformation because antagonists bind with up to 320-fold decreased affinity. None of the thermostabilising mutations are in the ZM241385 binding pocket, suggesting that the mutations affect ligand binding by altering the conformation of the receptor rather than through direct interactions with ligands. A_2A_R-GL26 shows considerable stability in short-chain detergents, which has allowed its purification and crystallisation.

## Graphical abstract

**Figure:**
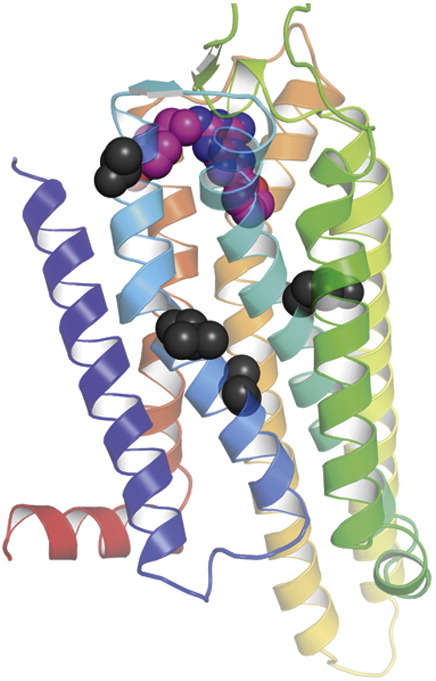

## Introduction

G-protein-coupled receptors (GPCRs) are the largest superfamily of transmembrane receptors with more than 800 members found in humans.^1^ They are key proteins in human physiology because they are the receptors for a wide variety of signalling molecules (agonists) such as hormones, neurotransmitters, lipids and nucleotides. Agonist binding to GPCRs causes a conformational change that allows coupling of either G proteins or β-arrestin and their subsequent activation,^2,3^ resulting in increased concentrations of intracellular second messengers such as cAMP and Ca^2+^. The pivotal role of GPCRs in intercellular communication makes them important targets for the development of drugs.^4,5^ Understanding how different classes of ligands (e.g., agonist, partial agonist, or inverse agonist) bind to receptors and how they affect the conformation of the receptor has been the goal of research for many decades. Recent successes in the structure determination of hormone-binding GPCRs have identified how antagonists are recognised by the receptors.^6–11^ In addition, recent structures of the β_1_ adrenoceptor (β_1_AR) and β_2_ adrenoceptor bound to agonists have given us the first insights^12–14^ into how agonists increase the probability of a receptor being in the R⁎ state (G-protein-coupling conformation) as opposed to the inverse agonist state R (G proteins unable to be activated).

Extracellular adenosine influences cellular function throughout the body, particular during cellular stresses such as anoxia, but in the central nervous system, it functions as a ubiquitous neuromodulator.^15^ There are four adenosine receptors found in humans, A_1_, A_2A_, A_2B_ and A_3_, which belong to the GPCR family A. The A_1_, A_2A_ and A_2B_ receptors are well known for being inhibited by the antagonist caffeine.^16^ Adenosine receptors are implicated in the pathophysiology of many neurological disorders such as Parkinson's disease, Huntington's disease as well as ischemia (cerebral and cardiac) and inflammatory and immune diseases.^17,18^ The adenosine A_2A_ receptor (A_2A_R) is therefore a potential target for treating many diseases, with the most advanced drug, preladenant, currently in phase III clinical trials for the treatment of Parkinson's disease.^19^

A structure of A_2A_R has been determined with the inverse agonist ZM241385 bound.^8^ The structure consisted of a receptor T4 lysozyme (T4L) fusion protein crystallised in lipidic cubic phase. Intracellular loop 3 in GPCRs has variable length and may be flexible before coupling to the G protein. Engineering of T4L into this loop produced a chimaeric receptor, A_2A_-T4L, which retains its ability to bind both agonists and antagonists, although this modified receptor binds agonists with higher affinity compared to wild-type (WT) A_2A_R;^8^ thus, its conformation may be slightly biased towards the agonist-bound state. A_2A_-T4L was crystallised in the presence of the high-affinity inverse agonist ZM241385 to help stabilise the detergent-solubilised A_2A_R. With its pharmacology favouring agonist binding in comparison to the WT receptor yet being crystallised with a tightly bound inverse agonist, the exact state of the crystallised complex is therefore open to debate. Another problem is that when low-affinity agonists bind to GPCRs, the agonist–receptor complex is often less stable than the antagonist–receptor complex,^20^ making it more difficult to crystallise and produce well-diffracting crystals. Therefore, to obtain the structure of A_2A_R with an agonist bound, we have extensively stabilised A_2A_R in a specific agonist-binding conformation.

Conformational thermostabilisation is a strategy used to engineer a membrane protein so that it is sufficiently stable in short-chain detergent for crystallisation and structure determination. The strategy has been applied to the thermostabilisation of the β_1_AR,^21,22^ the neurotensin receptor (NTS1)^23^ and A_2A_R.^24,25^ In each instance, it was observed that, if the selection for thermostabilising mutations was performed with an antagonist, then the thermostabilised mutant was preferentially in an antagonist-binding conformation; similarly, selection with an agonist resulted in a receptor in the agonist-binding conformation. Since there are distinctive structural differences between the agonist- and the antagonist-binding conformations, it is of little surprise that different mutations are required to stabilise the two different states.^24^ Stabilisation of β_1_AR and A_2A_R in the antagonist-bound conformation allowed the crystallisation and structure determination of both receptors^9^ (Dore *et al*., unpublished data). In contrast, thermostabilisation with agonists has proved to be more difficult. In the case of NTS1, the receptor was clearly thermostabilised, but on purification, the receptor tended to aggregate.^23^ This was ascribed to the stabilisation of the receptor simultaneously in both a ligand-free conformation and an agonist-bound conformation, thus resulting in a mutant that can undergo changes in structure upon ligand binding rather than being locked in a single conformation. Here, we describe a modified strategy for the stabilisation of A_2A_R in an agonist-bound conformation. The stabilised receptor A_2A_R-GL26 is now extremely stable in short-chain detergents when the agonist 5'-N-ethylcarboxamidoadenosine (NECA) is bound, and this has allowed its purification in a monodisperse state and its subsequent crystallisation.

## Results

### Identification of thermostabilising point mutations in agonist-bound A_2A_R

**Fig. 1:**
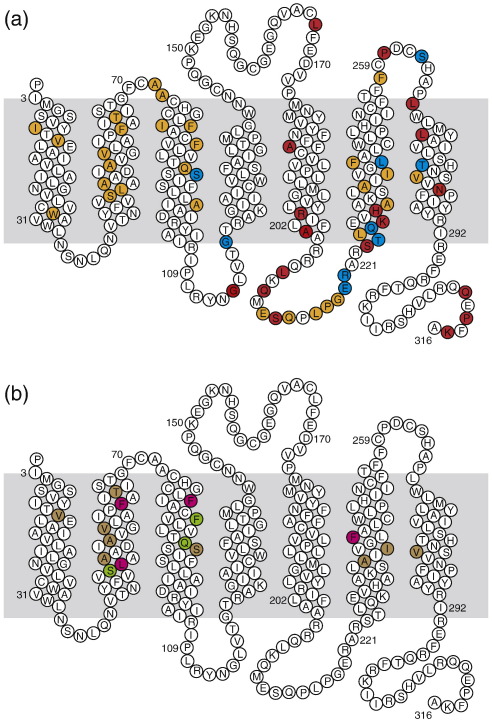
Positions of thermostabilising mutations in the primary sequence of human A_2A_R. The snake plot depicts the secondary structure elements found in the structure of A_2A_R, with the approximate position of the lipid bilayer shown in grey. (a) Thermostabilising mutations in the [^3^H]NECA-bound conformation are shown in orange. Mutations identified previously from [^3^H]NECA assays performed after heating the unliganded receptor are shown in red.^24^ Mutations that were selected by both assays are blue. (b) The 16 most thermostabilising mutations of the [^3^H]NECA-bound conformation of A_2A_R were re-assayed for thermostability in the antagonist-bound conformation using [^3^H]ZM241385 (Table 1): magenta, mutants that did not bind antagonist in this assay; green, mutants that are less stable than WT A_2A_R in the antagonist-bound conformation; and brown, mutants that are more stable than WT in the antagonist-bound conformation.

The strategy used to identify thermostabilising mutations involves creating a library of mutants throughout the receptor where every amino acid residue is changed to alanine or, if the residue is already alanine, to leucine. Each mutant receptor is then expressed and solubilised in detergent and has its thermostability determined by measuring the amount of receptor remaining after heating the sample for 30 min at a given temperature. A “thermostable” mutant in this context is therefore used to describe a mutated receptor with a decreased rate of unfolding, and the term thermostable is not meant to imply anything with respect to the thermodynamic properties of the receptor. In the previously published method^24^ to select mutations to produce a thermostable agonist-bound conformation of A_2A_R, the thermostability was determined by heating the detergent-solubilised receptor in the absence of ligand, quenching on ice and then performing a ligand binding assay using the agonist [^3^H]NECA. Thermostabilising mutations were identified (Fig. 1) and combined to construct the mutant A_2A_R-Rag23, which was 9 °C more thermostable than the WT receptor. Although significant, this small increase in stability was considered insufficient to guarantee success in structure determination. In the interim, it was found that heating the receptor in the thermostability assay in the presence of ^3^H-labeled agonist produced mutants with a greater increase in stability.^23^ Therefore, we rescreened all 315 point mutations made previously^24^ by heating the detergent-solubilised mutants in the presence of [^3^H]NECA (see Methods).

A necessary prerequisite to screen for [^3^H]NECA-bound themostabilising mutants was to define an optimum buffer to favour the formation of the receptor–agonist complex. The *K*_d_ for NECA binding to A_2A_R is reduced by high concentrations of detergent or NaCl, and the thermostability of [^3^H]NECA-bound A_2A_R is similarly reduced under these conditions.^24^ Therefore, the thermostability assay developed contained low concentrations of *n*-dodecyl-β-d-maltopyranoside (DDM) and no NaCl. After solubilisation in DDM, A_2A_R was bound to Ni^2+^-NTA resin, washed to reduce the DDM concentration and then eluted (see Methods). [^3^H]NECA was then added to this partially purified sample, which was then heated at various temperatures for 30 min and quenched on ice, and the receptor-bound ligand was separated from free ligand on a mini gel-filtration column.^26^ This assay was used to define the stability of the WT receptor (apparent *T*_m_) and for the initial screen of all the Ala/Leu scan mutants. We refer to this as the ligand plus format.^23^ The apparent *T*_m_ is defined as the temperature at which 50% of the solubilised receptor can still bind radioligand after a 30-min incubation.^21,23,24^ The apparent *T*_m_ for WT A_2A_R using the [^3^H]NECA thermostability assay (see Methods) is 28.6 ± 0.2 °C (*n* = 8).

**Fig. 2:**
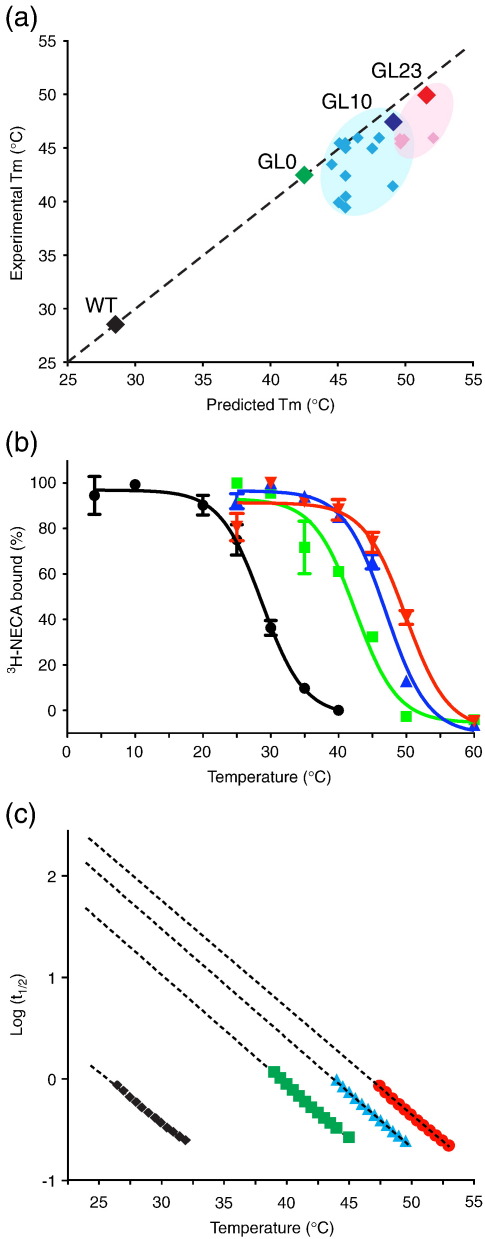
Thermostability of agonist-bound, DDM-solubilised A_2A_R and thermostabilised mutants. (a) Additive effect of thermostabilising mutations used to generate A_2A_R-GL23. Predicted *T*_m_ values were calculated by adding the Δ*T*_m_ for each mutation (Table 1) to the apparent *T*_m_ of WT A_2A_R. The broken line correlates with perfect additivity. WT A_2A_R is represented by a black diamond. The stabilities of the best thermostable mutants containing one, two or three point mutations are labelled, respectively, as follows: L48A (GL0), green diamond; L48A-Q89A (GL10), dark-blue diamond; and L48A-Q89A-T65A (GL23), red diamond. Other double mutants (light-blue diamonds) and triple mutants (light-red diamonds) that were less stable than the optimal combinations are also shown. (b) Thermostability assays were performed on receptors partially purified in 0.025% DDM and with [^3^H]NECA bound; A_2A_R (black circles), apparent *T*_m_ of 28.6 ± 0.2 °C, *n* = 8; GL0 (green squares), apparent *T*_m_ of 42.2 ± 1.0 °C, *n* = 5; GL10 (blue triangles), apparent *T*_m_ of 46.7 ± 0.4 °C, *n* = 3; and GL23 (red inverted triangles), apparent *T*_m_ of 49.9 ± 0.1 °C, *n* = 2. (c) Stability of mutants compared to WT A_2A_R based on *t*_1/2_ values calculated from (b) to allow the improvement in stability of the mutants compared to WT to be calculated: GL0, 49-fold; GL10, 136-fold; and GL23, 226-fold. The colour code is the same as in (b).

The library of Ala/Leu mutants made throughout A_2A_R^24^ was then expressed and solubilised in DDM, and a single-point thermostability assay was performed on each mutant by heating the [^3^H]NECA-bound receptor at 28 °C for 30 min; the results of these assays were compared to the thermostability of the WT receptor. Out of the 315 mutants screened, 38 were found to increase the thermostability of [^3^H]NECA-bound A_2A_R by a minimum of 40% and, in addition, maintained a minimum expression level of 30%, both values compared to WT A_2A_R (Fig. 1). The mutations thermostabilising [^3^H]NECA-bound A_2A_R are, in general, different from the mutants described previously that stabilise the ligand-free A_2A_R selected with [^3^H]NECA, although nine mutations are common to both experiments (Fig. 1). The apparent *T*_m_ was then determined for each of the 38 mutants, and the 16 most thermostabilising mutations (Table 1) were selected for further study. The L48A mutant provided the greatest thermostabilisation of the [^3^H]NECA-bound A_2A_R, conferring a 13.6 °C improvement in stability (apparent *T*_m_ of 42.2 ± 0.75 °C; Fig. 2); the Ballesteros–Weinstein numbers for all the amino acid residues discussed are presented in Table 1. The remaining 15 mutants improved the thermostability of [^3^H]NECA-bound A_2A_R by 2–6 °C (Table 1). The 16 selected mutations are highly clustered in the primary amino sequence of A_2A_R, with 7 mutations in transmembrane region (TM) 2, 4 in TM3 and 3 in TM6, with only 1 mutation in TM1 and TM7 (Fig. 1).

The thermostabilising mutants were then tested for their ability to thermostabilise A_2A_R when the inverse agonist ZM241385 was bound. In theory, if a mutation alters the equilibrium between R and R⁎ so that the agonist-binding conformation R⁎ is preferentially populated, then both the affinity for an inverse agonist and the thermostability of the receptor–inverse agonist complex could potentially be compromised. The apparent *T*_m_ of [^3^H]ZM241385-bound A_2A_R was 32.0 ± 0.1 °C (Table 1), which is 3.5 °C more stable than that of [^3^H]NECA-bound A_2A_R measured under identical conditions. The 15 mutants that showed the highest thermostabilities in the NECA-bound conformation were tested for thermostability when ZM241385 was bound. The results show that these 15 mutants can be categorised into three classes (Table 1). The first category contained mutants L48A, F62A, F79A and F242A, which did not bind [^3^H]ZM241385 at 4 °C in this experiment, suggesting a dramatic loss of affinity (see below for ligand binding experiments), and their thermostability with [^3^H]ZM241385 bound was not determined. The second category contained mutants S47A, F83A and Q89A, which stabilised the NECA-bound conformation and destabilised the ZM241385-bound conformation. The third group is composed of the remaining mutations that stabilised both agonist- and inverse-agonist-bound conformations (A50L, A54L, V57A, T65A, S90A, A236L, I238A and V282A).

### Combining mutants to make the optimally thermostable agonist-bound receptor A_2A_R-GL26

Given the unusually large increase in thermostability observed for A_2A_R-L48A, it was used as the starting point for the construction of an optimally stable agonist-binding receptor for structural studies, and it was renamed GL0. It has not usually proved possible to predict whether the combination of two thermostabilising point mutations will result in an additive effect on the thermostability or whether they will combine to destabilise the receptor. Therefore, 13 of the remaining thermostabilising point mutations were individually combined with the L48A mutation (Table 2). Previous experience of combining thermostabilising mutations has shown that mutants close to each other in the primary amino acid sequence were often not additive;^21,23,24^ thus, the double mutants of L48A with either S47A or A50L were not made. Each of the double mutants was expressed, and its thermostability was measured and compared to the predicted *T*_m_ [Δ*T*_m_ for each single mutant (Table 2) summed with the apparent *T*_m_ for WT A_2A_R]. Five of the double mutants did not increase thermostability compared to the L48A single mutant (Fig. 2). All of the remaining double mutants showed higher thermostabilities compared to GL0, with the best combination being L48A-Q89A (A_2A_R-GL10) with an apparent *T*_m_ of 46.7 ± 0.4 °C (Fig. 2). This strategy was repeated using the double mutant GL10 as the starting point and adding the five mutations found to be additive in the previous round. As previously mentioned, mutating residues close to each other often does not have an additive effect; thus, the S90A mutation was not tested, and in addition, GL10 containing the mutation F83A could not be made. One mutant showed an additive effect for the third mutation, L48A-Q89A-T65A (A_2A_R-GL23), which had an apparent *T*_m_ of 49.9 ± 0.1 °C (Fig. 2). It is interesting to note that all the mutations used to construct GL23 are in TM2 and TM3 and that none of the mutations in TM6 were additive with L48A (Fig. 1 and Table 2).

**Fig. 3:**
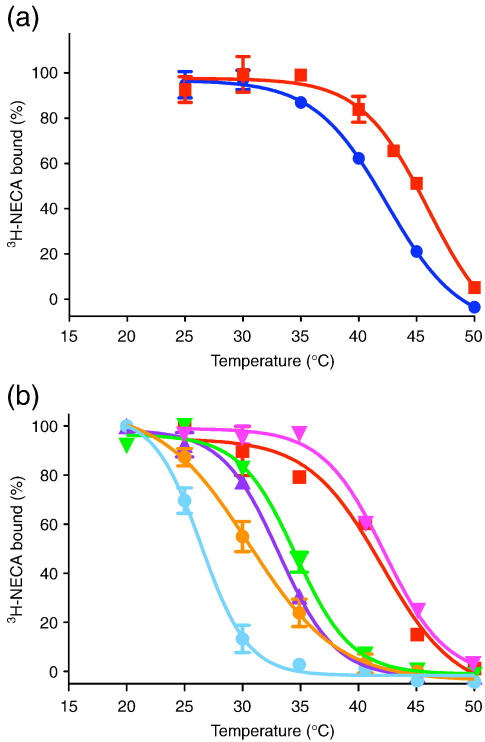
Thermostability of the mutants GL23 and GL26. (a) Thermostability of GL26 (red squares; apparent *T*_m_ of 44.5 ± 0.8 °C, *n* = 3) and GL23 (blue circles; apparent *T*_m_ of 42.2 ± 0.3 °C, *n* = 2) after partial purification in DM (0.17%), both with [^3^H]NECA bound. (b) The thermostability of GL26 with [^3^H]NECA bound was determined by partially purifying the receptor in different detergents. GL26 was solubilised in DM and immobilised on Ni^2+^-NTA agarose, and then detergent exchange was performed. The results are from a single experiment performed in triplicate, with the final concentration of detergent indicated: 0.39% decanoyl-*N*-hydroxyethylglucamide (pink inverted triangles), apparent *T*_m_ of 42.3 °C; 0.17% decylmaltoside (red squares), apparent *T*_m_ of 42.0 °C; 0.3% NG (green inverted triangles), apparent *T*_m_ of 34.6 °C; 0.52% foscholine-10 (blue triangles), apparent *T*_m_ of 33.1 °C; 0.42% octylthioglucoside (orange circles), apparent *T*_m_ of 30.5 °C; 0.37% polyoxyethylene C8E4 (pale blue circles), apparent *T*_m_ of 26.4 °C.

To improve the thermostability of GL23 further, we changed the detergent used in the thermostability assay to a shorter-chain detergent, *n*-decyl-β-d-maltopyranoside (DM). As expected, reducing the size of the detergent decreased the stability of the receptor, with A_2A_R-GL23 being 7.7 °C less stable in DM than in DDM (Table 2). After a last round of mutagenesis combining the additive mutants to GL23, it was found that the addition of A54L to make mutant GL26 yield the highest thermostability. A_2A_R-GL26 displayed very good thermostability in DM (apparent *T*_m_ of 44.5 ± 0.8 °C) and also excellent stability in a variety of other short-chain detergents ideal for crystallography (Fig. 3). A_2A_R-GL26 is 21.5 °C more stable than the WT receptor in DM, which has an apparent *T*_m_ of 23 °C when solubilised in this detergent.^24^

An alternative method for displaying the thermostability of the various mutants is shown in Fig. 2c, which relates the logarithm of the rate of inactivation of the receptor to the temperature (see Methods). Provided that all the assays are performed under identical conditions, this can be used to estimate the factor by which a receptor is stabilised compared to the WT receptor. Data extracted from Fig. 2b were therefore replotted in Fig. 2c and used to estimate that the rate of unfolding of NECA-bound A_2A_R-GL23 was about 230 times slower than that of the NECA-bound WT A_2A_R, implying a 230-fold improvement in the thermostability of A_2A_R-GL23.

### Pharmacological characterisation of thermostabilised mutants

**Fig. 4:**
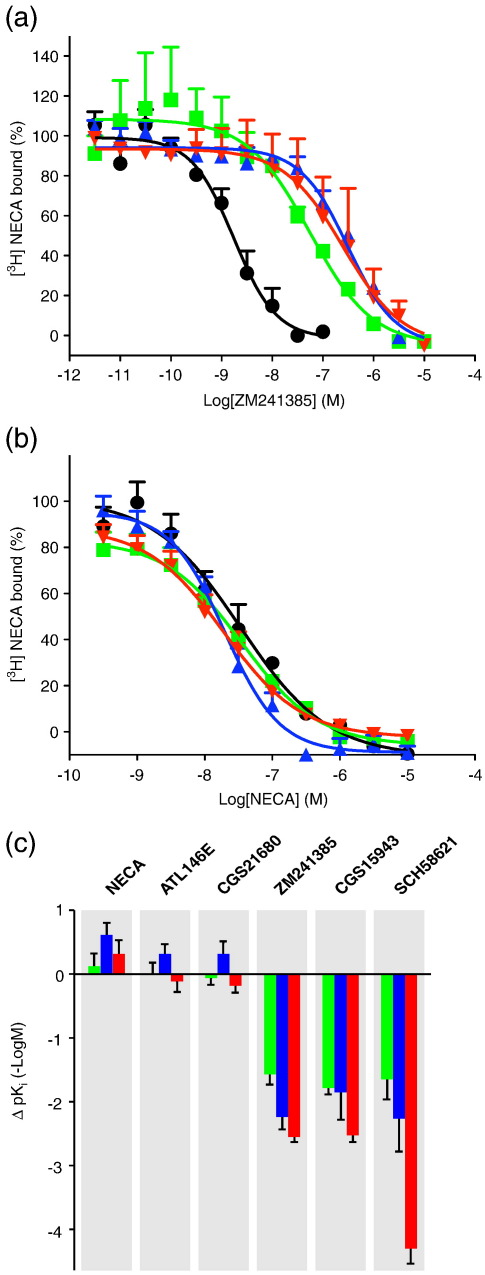
Affinities of agonists and antagonists for A_2A_R and the thermostabilised mutants. (a and b) Competition binding experiments were performed by measuring the displacement of [^3^H]NECA bound to receptors in CHO cell membranes. Experiments were performed using three agonists (NECA, ATL146e and CGS21680) and two antagonists (CGS15943 and SCH58621) and the inverse agonist ZM241385 with example curves shown for ZM241385 (a) and NECA (b); WT A_2A_R, black circles; GL0, green squares; GL23, blue triangles; and GL26, red inverted triangles. Full data are shown in Table 3. (c) The differences in affinities (Δp*K*_*i*_) between the WT A_2A_R and each of the mutants for the ligands tested were calculated from the p*K*_*i*_ values determined in Table 3; GL0, green; GL23, blue; and GL26, red.

Three of the mutants (GL0, GL23 and GL26) were transiently expressed in Chinese hamster ovary (CHO) cells; the membranes were purified; and competition binding analyses were performed using agonists, an inverse agonist and antagonists, in addition to saturation binding experiments performed with [^3^H]NECA, to characterise how the thermostabilising mutations have affected the conformation of A_2A_R (Table 3). The changes in p*K*_*i*_ for each mutant and all ligands tested are summarised in Fig. 4. All the mutants bound the antagonists CGS15943 and SCH58621, as well as the inverse agonist ZM241385, more weakly (Fig. 4). In contrast, there was no significant change in affinity for the binding of all the agonists tested (NECA, ATL146e and CGS21680) (Fig. 4) except that NECA bound three times more tightly to GL23 than to WT A_2A_R (Table 3). The data show that the major influence on the conformation of the ultimate mutant A_2A_R-GL26 is from the L48A mutation in GL0. This single point mutation accounted for 60–88% of the reduction in antagonist affinity observed in GL26.

### Purification of the thermostable mutant A_2A_R-GL31

**Fig. 5:**
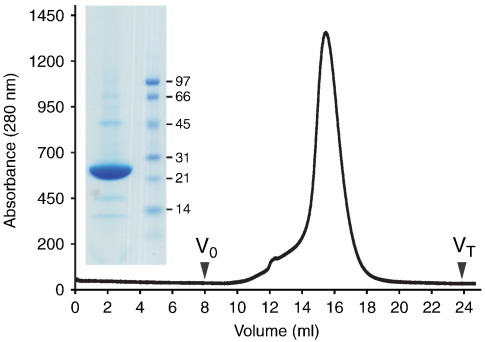
Purification of A_2A_R-GL31. The unglycosylated mutant of A_2A_R-GL26, A_2A_R-GL31, was expressed in insect cells using a recombinant baculovirus and purified on Ni^2+^-NTA. The receptor was further purified by size-exclusion chromatography (*A*_280_ trace is shown; void (*V*_0_) and total (*V*_T_) column volumes are indicated); this gave a symmetrical peak, which indicated that the preparation was monodisperse and homogenous. A Coomassie-blue-stained SDS-polyacrylamide gel (inset) showed that A_2A_R-GL31 represented a single band on the gel (left-hand lane) that was sufficiently pure for crystallisation (molecular weight markers are shown on the right).

A_2A_R-GL26 was expressed using the baculovirus expression system in insect cells to give about 2–3 mg/L of cell culture and then purified using a two-step process, a Ni^2+^-NTA column followed by size exclusion chromatography. However, purified GL26 consisted of two species differing in molecular mass by about 3 kDa, which is consistent with only a proportion of the receptor being N-glycosylated (data not shown). Therefore, the predicted N-glycosylation site Asn154 was mutated to Ala to make A_2A_R-GL31 (L48A-Q89A-T65A-A54L-N154A). A_2A_R-GL31 was expressed in insect cells and purified on a Ni^2+^-NTA column, followed by size-exclusion chromatography (Fig. 5). Even if the size-exclusion column was run using the detergent nonylglucoside (NG), purified A_2A_R-GL31 exhibited a symmetrical peak, which is indicative of a highly purified, monodisperse sample (Fig. 5). After concentration, one preparation yielded 1 mg of purified receptor from a 2-L culture, and the receptor could be concentrated up to 20 mg/mL without significant aggregation. A_2A_R-GL31 bound to NECA produced good-quality crystals that diffracted isotropically to 2.6 Å resolution (Lebon *et al.*, unpublished data).

## Discussion

Conformational thermostabilisation of GPCRs has proven to be a successful strategy for their structure determination when the receptor is stabilised in the antagonist state, with structures of both a β_1_AR^9^ and an A_2A_R (Doré *et al*. unpublished data) with antagonists bound having been determined. The real value of the approach was recently highlighted by the structures of β_1_AR bound to low-affinity agonists.^14^ The other approaches that have also given GPCR structures^27^ rely on increasing the hydrophilic area of the receptor by binding an antibody fragment and/or with a T4L fusion and on the thermostabilisation of the receptor by formation of a complex with a high-affinity ligand with a slow off-rate.^8,28^ In the case of A_2A_R, we would like to determine its structure bound to its natural agonist, adenosine; thus, thermostabilisation seemed to be the logical approach to take. Two different thermostabilisation procedures have been developed previously for stabilising a GPCR in an agonist-bound conformation, and these gave rise to thermostable mutants A_2A_R-Rag23^24^ and NTS1-7m,^23^ but in both cases, the degree of thermostabilisation attained was probably too small to guarantee the formation of well-diffracting crystals. Here, we describe another strategy that produced the mutant A_2A_R-GL26, which is highly thermostable and has already been purified and crystallised. The two significant changes introduced here in relation to previous procedures were, firstly, the use of [^3^H]NECA-bound A_2A_R in the thermostability assay and, secondly, the removal of the requirement for simultaneous stabilisation of the unliganded and NECA-bound conformations. The resulting mutant, A_2A_R-GL26, is similar in stability upon detergent solubilisation to native rhodopsin^29^ or β_1_AR-m23,^21^ both of which have been crystallised and their structures determined to high resolution.

Four mutations were required to thermostabilise A_2a_R in the agonist-bound conformation. The single mutation with the greatest thermostabilising effect was L48A, which increased the stability of NECA-bound A_2A_R by 13.6 °C. The L48A mutant was therefore an obvious candidate to start the thermostabilisation of the A_2A_ NECA-bound conformation. The strategy was to make a series of double mutants by adding the best thermostabilising mutants to L48A, testing their thermostability, picking the most thermostable double mutant and then adding the next best single thermostabilising mutation. At each stage, only those single mutations that gave a clear additive increase in thermostability were tested in the next round. Out of 13 double mutants constructed and tested for their thermostability, the L48A-Q89A mutant had the highest thermostability, which was only slightly lower than the *T*_m_ predicted by adding the Δ*T*_m_ for Q89A to the *T*_m_ for L89A. In a similar fashion, T65A was found to further thermostabilise the receptor to make the triple mutant GL23 (L48A-Q89A-T65A) with an apparent *T*_m_ of 50 °C in DDM. We have sometimes found that the measurement of apparent *T*_m_ at high temperatures may be less accurate than desired due to nonspecific protein aggregation in the sample; therefore, we decided to carry out one more round of thermostabilisation in DM. As expected, the thermostability of GL23 was lower in DM than in DDM (Fig. 3). Among the five mutations tested, the best additive effect was observed for the A54L mutant. The final mutant GL26 displays an apparent *T*_m_ of 44.5 °C in 0.15% DM, which is similar to that observed for the thermostable mutant β_1_AR-m23 (apparent *T*_m_ of 48 °C).^21^ A_2A_R-GL26 also displayed considerable stability in relatively harsh detergents such as foscholine-10 (FC10), decanoyl-*N*-hydroxyethylglucamide and *n*-octyl-β-d-thioglucopyranoside, which have been used previously to crystallise β_1_AR-m23.^30^

In previous thermostabilisation experiments, each receptor was stabilised in a particular conformation depending on whether an agonist or an antagonist was used for the selection of thermostable mutants. The conformation of A_2A_R-GL26 was therefore assessed by performing ligand binding assays using both agonists and antagonists. The binding affinities for the inverse agonist ZM241385 and the antagonist CGS15943 were reduced by 320-fold, whereas the only statistically significant change in agonist binding affinity was observed for NECA (3-fold increase). The affinities for ATL146e and CGS21680 remained similar to those of WT A_2A_R. None of the mutations used to thermostabilise A_2A_R-GL26 are in the ligand binding pocket, which suggests that they acted by affecting the global conformation of the receptor. It is not possible to define exactly which conformational state A_2A_R-GL26 is in, except by determining its structure, but the binding data are consistent with A_2A_R-GL26 being in an agonist-binding conformation. However, it is very unlikely that the conformation is identical with the fully activated state because it would then be expected that agonist affinity would be increased by a factor of 15–40 or more.^31,32^ It is thus anticipated that the receptor will represent a conformation along the activation pathway, between the R state and the R⁎ state.

**Fig. 6:**
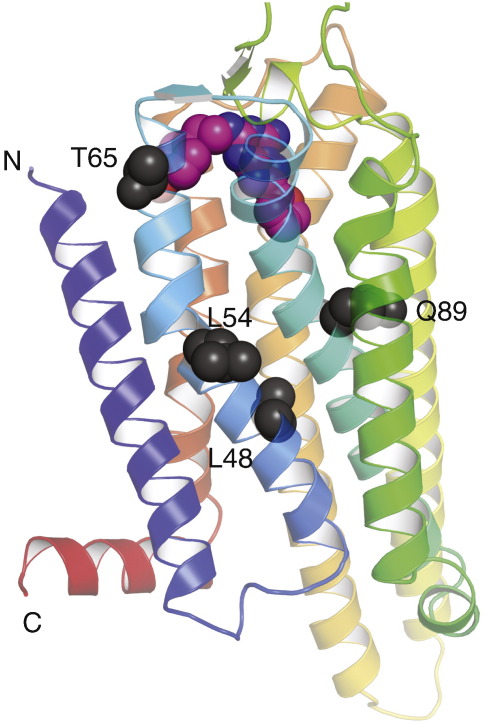
Positions of the thermostabilising mutations in the antagonist-bound A_2A_ structure. The structure of A_2A_R-StaR2 (Protein Data Bank code 3PWH) thermostabilised in an antagonist-bound conformation is shown in rainbow colouration with the N-terminus and C-terminus labelled (N and C, respectively) and the bound antagonist ZM241385 depicted as a space-filling model (C, pink: N, blue; and O, red). The four amino acid residues mutated in A_2A_R to generate the thermostable mutant GL26 (L48A, Q89A, T65A and A54L) are depicted as space-filling models (dark grey). Note that the amino acid sequence shown is that of A_2A_R-StaR2, which also contains the A54L thermostabilising mutation,^25^ whereas Leu48, Gln89 and Thr65 are identical with WT A_2A_R.

Analysis of the ZM241385-bound structure of A_2A_R^8^ showed that none of the thermostabilising mutants in A_2A_R-GL26 make direct contact to the ligand (Fig. 6). It is therefore likely that the introduction of the four thermostabilising mutations L48A, T65A, Q89A and A54L has induced a conformational change in the receptor, and the binding data support the view that the mutant is in an agonist-binding conformation between R and R⁎. The single biggest effect on ligand binding was seen for the mutation L48A^2.46^ (superscript refers to the Ballesteros–Weinstein numbering system^33^) with a 2-fold increase in the affinity for the agonist NECA and a 50-fold decrease in affinity for the inverse agonist ZM241385. In the entire GPCR family, Leu^2.46^ is one of the most conserved residues in TM2 (approximately L 96%, M 2%, I 1.5% and V/T 0.5%). When mutated to alanine, Leu^2.46^ displays constitutive activity in rhodopsin^34^ and the thyrotropin receptor.^35^ The side chain of Leu48^2.46^ is located near the cytoplasmic end of TM2 and oriented towards the core of the receptor helix bundle close to the NPXXY motif in TM7, which is composed of the highly conserved residues Asn^7.49^, Pro^7.50^ and Tyr^7.53^. Leu^2.46^ has been described as being involved in a hydrophobic interaction with Asn^7.49^, which may constrain the receptor in an inactive conformation,^35^ and was suggested to stabilise the ground state of rhodopsin.^34^ The Q89A mutation was previously reported to increase the affinity of agonists and decrease the affinity for antagonists, perhaps through an indirect effect on the receptor,^36^ which we also observed here (Fig. 4). In the crystal structure of A_2A_R bound to ZM241385, both T65A and A54L are located in TM2 facing the lipid bilayer; thus, it is unclear why these mutations are thermostabilising.

The rationale for thermostabilising membrane proteins is to allow the use of short-chain detergents during both purification and crystallisation, which will improve the probability of success in obtaining well-diffracting crystals that are suitable for structure determination.^37^ Therefore, the success of any thermostabilisation procedure should be apparent during purification of the mutated receptor because it should not aggregate even if relatively harsh detergents are used. Initial purification of A_2A_R-GL26 showed that the receptor was present as both an unglycosylated and an N-glycosylated product (results not shown); thus, the additional mutation N154A was introduced into A_2A_R-GL26 to make the non-glycosylated mutant A_2A_R-GL31. This was subsequently purified in NG, and the product was monodisperse and pure, showing that the protein was ideal for crystallography. Indeed, crystals were obtained relatively easily and have been improved to diffract to better than 2.6 Å resolution; the structure of A_2A_R-GL31 is currently under refinement. Thus, the simplified thermostabilisation strategy presented here for stabilising the agonist-bound form of GPCRs was successful and should be equally applicable to other GPCRs.

## Methods

### Expression of adenosine A_2A_R point mutants in *Escherichia coli*

The library of Ala/Leu scan mutants in the receptor A_2a_R-(2–316) was expressed from plasmid pRG/III-hs-MBP in *E. coli* strain DH5α as previously described.^24^ Cells were grown at 37 °C in 2-L flasks containing 500 mL of 2× tryptone–yeast medium supplemented with ampicillin (100 μg/mL) and glucose (0.2% w/v). At an OD_600_ of 0.7, IPTG and theophylline were added at final concentrations of 0.5 mM and 100 μM, respectively, and the temperature was reduced to 20 °C. After 22 to 24 h, cells were harvested in aliquots of 14 mL, centrifuged (30 min, 5000***g***) and stored at − 20 °C.

### Solubilisation and partial purification of adenosine A_2A_R mutants for thermostability assays

An aliquot of *E. coli* cells (14 mL) was thawed on ice and resupended in 500 μL of buffer A [50 mM Tris–HCl (pH 7.4), 0.4 M NaCl, 250 μg/mL lysozyme (Sigma) and 1 mg/mL DNase I (Sigma), supplemented with complete ethylenediaminetetraacetic acid (EDTA)-free Protease inhibitor cocktail (Roche)] and incubated for 1 h at 4 °C. Samples were then sonicated for 1 min at 4 °C using a cup-horn sonicator. The receptors were solubilised by adding 1% DDM and incubated for 1 h at 4 °C. Insoluble material was removed by centrifugation (5 min, 13,000***g***, 4 °C). The solubilised receptors were partially purified with Ni-NTA agarose (Qiagen). Agarose beads (300 μL) pre-equilibrated in buffer A were added to 700 μL of solubilised receptor. To reduce the detergent concentration by dilution, we added a solution of 50 mM Tris–HCl, pH 7.4, and 0.4 M NaCl to a final volume of 2 mL. After 2 h of incubation at 4 °C, samples were centrifuged (13,000***g***, 10 s, 4 °C), washed three times in buffer B (25 mM Hepes, pH 7.4, and 0.025% DDM) and then eluted in buffer B supplemented with 50 mM histidine for 30 min at 4 °C. The supernatant was used directly in radioligand binding assay.

### Radioligand binding assay and thermostability assay for detergent-solubilised receptors

Solubilised receptor (108 μL) was mixed with 12 μL of 4 μM [^3^H]NECA (final concentration of 400 nM) or 12 μL of 1 μM [^3^H]ZM241385 (final concentration of 100 nM). Radioligand concentrations used were approximately 10-fold the *K*_d_ value. The sample was incubated for 45 min at 4 °C, then 30 min at the specified temperature and then 30 min at 4 °C. Receptor-bound and free radioligands were separated as previously described on mini gel-filtration columns.^26^ Receptor-bound ligands were transferred to a 96-well plate (PerkinElmer) and mixed with 200 μL of Optiphase supermix (PerkinElmer). The bound ^3^H-labeled ligand was determined using a 1450 Microbeta Trilux counter (1 min per sample).

### Mammalian cell culture and receptor expression

CHO cells were maintained in culture in Dulbecco's modified Eagle's medium/HAMs F12 media containing 10% fetal bovine serum (FBS). Cells were transfected with either WT adenosine A_2A_R or a stabilised receptor construct using GeneJuice according to the manufacturer's instructions. After 48 h post-transfection, cells were harvested by scraping and centrifuged (200***g***, 5 min, 4 °C). The supernatant was removed, and the pellet was resuspended in 10 mL of 20 mM Hepes buffer + 10 mM EDTA buffer (pH 7.4). The membrane suspension was homogenised (10 s, 20,500 rpm) and centrifuged (200***g***, 15 min, 4 °C). The supernatant was collected, the pellet was resuspended in 10 mL of Hepes/EDTA buffer and the solution was homogenised and centrifuged as described before. The collected supernatant was centrifuged (30 min, 40,000***g***, 4 °C). Pellets were resuspended in 20 mM Hepes, pH 7.4, and 0.1 mM EDTA to a concentration of 1 mg/mL and stored at − 80 °C until further use.

### Ligand binding assays, saturation and competition binding experiment

Membranes from CHO cells transiently expressing receptors (10–15 μg/well) were assessed using competition [^3^H]NECA binding in buffer containing 50 mM Tris–HCl (pH 7.4). Nonspecific binding was defined using 1 μM CGS21680. After 1 h of incubation at 25 °C, assays were terminated by filtration through 96-well GF/B filter plates presoaked with 0.1% polyethyleneimine and washed with 5 × 0.5 mL water. Plates were dried, and bound ligand was measured using a Microbeta counter. Inhibition curves were fitted to a four-parameter logistic equation to determine IC_50_ values, which were converted into *K*_*i*_ values using *K*_d_ values determined by saturation binding and the [^3^H]NECA concentration (∼ 10 nM).

### Purification of NECA-bound A_2A_R-GL31

Receptors were expressed with the baculovirus system using cells derived from *Trichoplusia ni* (High 5™) and the vector pBacPAK8 (Invitrogen). Insect cells were grown in suspension in a maximum volume of 500 mL in 2-L roller bottles (Corning) at 27 °C with shaking at 150 rpm. Sf9 cells were grown in TNM-FH medium supplemented with 10% FBS, and Tni cells were grown in EXcell 405 medium supplemented with 5% FBS (heat inactivated); all media were supplemented with 1% lipids (Invitrogen). The GL31 construct was inserted into plasmid pBacPAK8 using the restriction enzyme BamHI/XbaI*.* Sf9 cells were used to generate the first virus passages and to obtain second- and third-passage high-titre virus stocks. Tni cells were grown to 2 × 10^6^ to 2.5 × 10^6^ cells/L, diluted in a 1:1 volume ratio with fresh media and infected with the recombinant baculovirus. Cells were harvested 72 h postinfection, resuspended in 25 mM Hepes, pH 7.4, and 1× protease inhibitor cocktail and snap-frozen in liquid N_2_.

All protein purification steps were performed at 4 °C. Frozen cell pellets equivalent to 2 L of cell culture were thawed and resuspended at room temperature in 25 mM Hepes (pH 7.4), EDTA 1 mM, PMSF (0.5 mM), pepstatin (1 μg/mL) and leupeptin (1 μg/mL) or 1× protease inhibitor cocktail to give a final volume of 360 mL. The cells were centrifuged (120,000***g***, 2 h, 4 °C) to pellet the washed cells and membranes, the supernatant was carefully removed and the pellet was resupended in 240 mL of the same buffer. Cells were homogenised using a Polytron (12,000 rpm, 2 × 15 s on ice) and centrifuged to pellet the membranes (45,000***g***, 2 h, 4 °C). The pellet was resuspended in 25 mM Hepes (pH 7.4), PMSF (0.5 mM), pepstatin (1 μg/mL) and leupeptin (1 μg/mL) or 1× protease inhibitor cocktail, homogenised using a Polytron (12,000 rpm, 2 × 15 s on ice) and snap-frozen in liquid N_2_.

Membranes were thawed at room temperature, diluted with 25 mM Hepes (pH 7.4), PMSF (0.5 mM), pepstatin (1 μg/mL) and leupeptin (1 μg/mL) or 1× protease inhibitor cocktail (100 mL). Membranes were pre-incubated with NECA at 100 μM for 45 min before solubilisation. Receptors were solubilised by adding DM and NaCl to give final concentrations of 1.5% and 0.3 M, respectively, followed by centrifugation (120,000***g***, 45 min, 4 °C). The solubilised receptor sample was then filtered through a 0.22-μm filter (Millipore) and applied at 0.3 mL/min to a 5-mL Ni-NTA superflow cartridge (Qiagen) pre-equilibrated with buffer [25 mM Hepes (pH 7.4), 0.1 M NaCl, 100 μM NECA, 0.15% DM and 2.5 mM imidazole]. The column was washed (1 mL/min) with the same buffer supplemented with 10, 40 or 80 mM imidazole for 5, 10 and 5 column volumes, respectively, and then eluted with 5 column volumes of elution buffer [25 mM Hepes (pH 7.4), 0.1 M NaCl, 100 μM NECA, 0.15% DM and 250 mM imidazole]. The eluted receptor was mixed with tobacco etch virus protease to cleave the tag for 4–6 h at 4 °C. After cleavage, 14–16 mL of the pooled fractions was concentrated to 2 mL using an Amicon-ultra spin concentrator (Ultracel-50K; Millipore) and loaded onto a PD-10 column (GE Healthcare) in order to remove the imidazole. A negative purification was used to remove the tobacco etch virus protease by loading the sample in batch onto 5 mL Ni-NTA (Qiagen) pre-equilibrated in 25 mM Hepes (pH 7.4), 0.1 M NaCl, 100 μM NECA, 0.15% DM and 40 mM imidazole and incubated for 30 min. The resin was spun down, and the supernatant containing the receptor was removed. For detergent exchange (into, e.g., 0.35% NG), the sample (5.5–6 mL) was concentrated down to 0.5 mL using an Amicon-ultra concentrator (Ultracel-50K; Millipore), diluted 10-fold in 25 mM Hepes (pH 7.4), 0.1 M NaCl, 100 μM NECA and 0.35% NG and concentrated down again to 0.3 to 0.5 mL. The protein sample was applied to a 10/30 S200 size-exclusion column pre-equilibrated in 25 mM Hepes (pH 7.4), 0.1 M NaCl, 100 μM NECA and 0.35% NG and run at 0.5 mL/min. Protein determination was performed using the amido black assay.^38^

### Determination of receptor half-life (*t*_1/2_) using the Arrhenius law

For all the thermostabilisation studies carried out in our laboratory, the apparent *T*_m_ is the temperature for which 50% of the solubilised receptor remains folded after 30 min of incubation. The rate constant of the protein denaturation is a function of the temperature, and consequently, thermal denaturation of a protein can be compared to a chemical reaction for which the rate of the reaction is dependant on the temperature. Arrhenius established that any chemical reaction is temperature dependant. We used a simplified version of the Arrhenius law to estimate the *t*_1/2_ of A_2A_R: *A* = *A*_0_exp(− 0.693 × *t*/*t*_1/2_), in which *A* is the total sample activity, *A*_0_ is the activity for a defined temperature, *t* is the experimental time used for heating the sample and *t*_1/2_ is the half-life of A_2A_R bound to its agonist NECA. From the experimentally determined *T*_m_ curves, the values *t* and *A* were selected as being the slope of the curve, defining a window around the *T*_m_ value, which represents the linear part of the curve, from 26.5 °C to 32 °C for the WT, from 39 °C to 45 °C for GL0, from 44 °C to 50 °C for GL10 and from 47.5 °C to 53 °C for GL23. The reference measurement representing the total binding activity of the sample *A*_0_ was considered to be measured at 4 °C. From the graphical representation *t* = Log_10_(*t*_1/2_) (Fig. 2c), we could extract a half-life for each construct at any temperature to define the improvement in stability of the various mutants compared to WT A_2A_R.

## Figures and Tables

**Table 1:** Thermostability of A_2A_R mutants with either agonist or antagonist bound

A_2A_R mutation	Ballesteros–Weinstein	Apparent *T*_m_ (°C)			
		Agonist		Antagonist	
		[^3^H]NECA	Δ*T*_m_	[^3^H]ZM241385	Δ*T*_m_
WT	—	28.5	—	32	—
V12A	1.38	30.5	+ 2	n.d.^a^	n.d.
S47A	2.45	31	+ 2.5	25.5	− 6.5
L48A	2.46	42.5	+ 14	0^b^	n.d.
A50L	2.48	31	+ 2.5	38.5	+ 6.5
A54L	2.52	33.5	+ 5	38	+ 6
V57A	2.55	34.5	+ 6	38	+ 6
F62A	2.60	31	+ 2.5	0	n.d.
T65A	2.63	33	+ 4.5	38	+ 6
F79A	3.27	31	+ 2.5	0	n.d.
F83A	3.31	30	+ 1.5	16.5	− 15.5
Q89A	3.37	34.5	+ 6	26	− 8
S90A	3.38	32	+ 3.5	32	0
A236L	6.38	31	+ 2.5	38	+ 6
I238A	6.40	31	+ 2.5	36	+ 4
F242A	6.44	31	+ 2.5	0	n.d.
V282A	7.47	30.5	+ 2	33	+ 1

Values were determined from a single thermostability curve with values determined in triplicate with an estimated error of ± 0.5 °C. These values were used to give a rank order of thermostabilisation for the different mutants. For final values for WT A_2A_R and L48A (GL0), please refer to Table 2.

a Not determined.

b No binding detected at antagonist concentration used.

**Table 2:** Combinations of mutants tested for thermostabilising the NECA-bound conformation of A_2A_R

	Mutant name	A_2A_R mutations	Apparent *T*_m_ in DDM (°C)		
			Predicted	Single measurement^a^	Final values^a^
WT A_2A_R	—	—	—	—	28.6 ± 0.2 (*n* = 8)
Single mutant	GL0	L48A	42.5	42.5	42.2 ± 1.0 (*n* = 5)
Double mutant	GL1	L48A-V12A	45	45.5	
	GL4	L48A-A54L	47.5	46	
	GL5	L48A-V57A	48.5	41.5	
	GL6	L48A-F62A	45	45	
	GL7	L48A-T65A	47	45	
	GL8	L48A-F79A	45	45.5	
	GL9	L48A-F83A	44	43.5	
	GL10	L48A-Q89A	48.5	47.5	46.7 ± 0.4 (*n* = 3)
	GL11	L48A-S90A	46	46	
	GL14	L48A-A236L	45	40.5	
	GL15	L48A-I238A	45	42.5	
	GL17	L48A-F242A	45	39.5	
	GL19	L48A-V282A	44.5	40	
Triple mutant	GL20	L48A-Q89A-V12A	49.5	46	
	GL21	L48A-Q89A-A54L	52	46	
	GL22	L48A-Q89A-F62A	49.5	45.5	
	GL24	L48A-Q89A-F79A	49.5	46	
	GL23	L48A-Q89A-T65A	51.5	50	49.9 ± 0.1 (*n* = 2)

			Apparent *T*_m_ in DM (°C)		

Predicted	Single measurement^a^	Final values^a^

Triple mutant	GL23	L48A-Q89A-T65A			42.2 ± 0.3 (*n* = 2)
Quadruple mutant	GL25	L48A-Q89A-T65A-A50L	46	44.5	
	GL26	L48A-Q89A-T65A-A54L	49	46	44.5 ± 0.8 (*n* = 3)
	GL27	L48A-Q89A-T65A-F83A	44.5	44	
	GL29	L48A-Q89A-T65A- S263A	44.5	44.5	

a Values were determined initially from a single thermostability curve, with measurements performed in triplicate, to find the most thermostable mutants; replicate experiments were performed only for key mutants. Predicted *T*_m_ was calculated from the parental experimental *T*_m_ value (single or double mutants) and the value of the Δ*T*_m_ for the single mutant tested. Experimental measurements were then compared with the predicted *T*_m_ and classified as (i) an additive effect when the experimental value is equal or similar to the predicted *T*_m_ (± 1.5 °C) and (ii) nonadditive when the experimental value is different from the predicted *T*_m_. Quadruple mutants were tested in DM.

**Table 3:** Comparison of affinities of agonist and antagonist binding to A_2A_R and thermostable mutants

		p*K*_*i*_ (− Log M)			
		WT	GL0	GL23	GL26
Agonist	NECA	7.82 ± 0.20	7.94 ± 0.06	8.43 ± 0.07⁎	8.13 ± 0.11
	ATL146e	7.95 ± 0.17	7.96 ± 0.01	8.25 ± 0.01	7.85 ± 0.08
	CGS21680	6.94 ± 0.11	6.89 ± 0.05	7.24 ± 0.19	6.77 ± 0.06
Antagonist	ZM241385	9.22 ± 0.04	7.65 ± 0.17⁎⁎⁎	6.89 ± 0.06⁎⁎⁎	6.67 ± 0.08⁎⁎⁎
	CGS15943	9.68 ± 0.11	7.91 ± 0.06⁎⁎⁎	7.49 ± 0.10⁎⁎⁎	7.17 ± 0.08⁎⁎⁎
	SCH58621	8.92 ± 0.17	6.97 ± 0.06⁎⁎⁎	7.10 ± 0.12⁎⁎⁎	4.61 ± 0.20⁎⁎⁎

Competition experiments were performed by displacement of [^3^H]NECA from receptors transiently expressed in CHO cells. The p*K*_*i*_ values are the mean of three independent experiments performed in triplicate ± standard error of the mean. p*K*_*i*_ values were calculated from the IC_50_ using the Cheng–Prusoff equation and the following values for *K*_d_ (nM) for [^3^H]NECA: WT, 13.45 ± 0.44; GL0, 6.39 ± 0.59⁎⁎⁎; GL23, 4.65 ± 0.67⁎⁎⁎; and GL26, 6.33 ± 0.66⁎⁎⁎. *P* values were determined using a one-way ANOVA with Dunnett's post-hoc test: ⁎, *P* < 0.05; ⁎⁎⁎, *P* < 0.001 with respect to the WT A_2A_R.
